# A Self-Adaptive Serious Game to Improve Motor Learning Among Older Adults in Immersive Virtual Reality: Short-Term Longitudinal Pre-Post Study on Retention and Transfer

**DOI:** 10.2196/64004

**Published:** 2025-03-03

**Authors:** Gauthier Everard, Louise Declerck, Thierry Lejeune, Martin Gareth Edwards, Justine Bogacki, Cléo Reiprich, Kelly Delvigne, Nicolas Legrain, Charles Sebiyo Batcho

**Affiliations:** 1 School of Rehabilitation Sciences Faculty of Medicine Laval University Québec, QC Canada; 2 Centre interdisciplinaire de recherche en réadaptation et intégration sociale Université Laval Québec, QC Canada; 3 Neuro Musculo Skeletal Lab (NMSK) Institut de Recherche Expérimentale et Clinique UCLouvain Brussels Belgium; 4 Services de Médecine Physique et Réadaptation Cliniques Universitaires Saint-Luc Brussels Belgium; 5 Louvain Bionics UCLouvain Louvain-la-neuve Belgium; 6 Psychological Sciences Research Institute (IPSY) UCLouvain Louvain-la-neuve Belgium

**Keywords:** virtual reality, aged, learning, upper extremity, video games, kinematics

## Abstract

**Background:**

Despite their potential, the use of serious games within immersive virtual reality (iVR) for enhancing motor skills in older adults remains relatively unexplored. In this study, we developed a self-adaptive serious game in iVR called REAsmash-iVR. This game involves swiftly locating and striking a digital mole presented with various distractors.

**Objective:**

This short-term longitudinal pre-post study aims to evaluate REAsmash-iVR’s efficacy in promoting motor learning in older adults. Specifically, we seek to determine the transfer and retention of motor learning achieved through REAsmash-iVR to other iVR tasks.

**Methods:**

A total of 20 older adults participated in the study, engaging with REAsmash-iVR over 7 consecutive days. The evaluation included iVR tests such as KinematicsVR and a VR adaptation of the Box and Block Test (BBT-VR). KinematicsVR tasks included drawing straight lines and circles as fast and as accurately as possible, while BBT-VR required participants to move digital cubes as quickly as possible within 60 seconds. Assessments were conducted before and after the intervention, with a follow-up at 1 week post intervention. The primary outcome focused on evaluating the impact of REAsmash-iVR on speed-accuracy trade-off during KinematicsVR tasks. Secondary outcomes included analyzing movement smoothness, measured by spectral arc length, and BBT-VR scores.

**Results:**

Results revealed significant improvements in speed-accuracy trade-off post intervention compared to that before the intervention, with notable retention of skills for straight lines (t_19_=5.46; *P*<.001; Cohen *d*=1.13) and circle drawing (t_19_=3.84; *P*=.001; Cohen *d*=0.787). Likewise, there was a significant enhancement in spectral arc length, particularly for circle drawing (*χ*²_2_=11.2; *P*=.004; ε^2^=0.23), but not for straight-line drawing (*χ*²_2_=2.1; *P*=.35; ε^2^=0.003). Additionally, participants demonstrated transfer with significant improvement (*q*=5.26; *P*<.001; Cohen *r*=0.678) and retention (*q*=6.82; *P*<.001; Cohen *r*=0.880) in BBT-VR skills.

**Conclusions:**

These findings provide perspectives for the use of iVR to improve motor learning in older adults through delivering self-adaptive serious games targeting motor and cognitive functions.

**Trial Registration:**

ClinicalTrials.gov NCT04694833; https://clinicaltrials.gov/study/NCT04694833

## Introduction

By 2050, the proportion of older adults (older than 65 years) worldwide will nearly reach 22% [1,2]. Within the population, some older adults experience progressive functional decline, encompassing both motor and cognitive aspects [3]. This decline exacerbates issues of inactivity and sedentariness [4] contributing to increased prevalence of age-related diseases [5,6]. As an illustration, it is projected that by the year 2050, the number of individuals affected by major neurocognitive disorders will surge from 50 to 152 million worldwide, marking a 3-fold increase in cases [7].

Older adults’ functional decline is typically associated with lower motor functions [8] and reduced quality of life [9,10]. Consequently, older adults tend to adopt compensatory behaviors that mitigate the impact of these reduced functions on their daily living activities [11]. These behavioral compensations include making slower, less accurate, less linear, and less smooth movements [12,13].

Motor learning refers to any experience-dependent improvement of a skill and typically involves both motor and cognitive processes [14]. Once learned, motor skills can be retained for an extended duration, resulting in sustained enhancements in performance [14]. A skill is not deemed fully acquired until the ability to retain or apply it in different contexts is demonstrated [15]. Research suggests that both healthy older adults and those with neurocognitive disorders can improve their motor performance through motor learning, exhibiting enhancements in movement speed, smoothness, coordination, and accuracy [16,17]. However, current motor learning programs mainly focus on gait and balance and therefore, demand time and availability from caregivers [18]. In response to these demands, new portable devices with cost-saving potential such as virtual reality (VR) might be of interest to promote motor learning in older adults.

VR can be defined as a computerized technological system that allows users to interact with a simulated multisensorial environment while providing real-time performance feedback [19]. Two main types of VR experience exist. Nonimmersive VR (niVR) is where users maintain awareness of their physical surroundings and receive visual feedback via a 2D display. Immersive VR (iVR) facilitates total immersion in the digital environment (using a head-mounted display or a large, curved screen with a panoramic view), with a comprehensive panoramic perspective [20]. Recent research suggests that VR programs may enhance participants’ level of physical activity [21] and cognitive skills such as reaction time [22].

In rehabilitation, VR devices are often combined with serious games. Serious games refer to any game-based initiative that primarily focuses on learning objectives (such as education or rehabilitation) rather than simple entertainment [23]. Serious games have the capability to fulfill motor learning principles [24-26], as they motivate participants to make numerous practice repetitions through the use of multisensory feedback, personalized challenges, and through use of compelling and enriched environments [27,28]. After a certain period of familiarization, VR devices may allow participants to follow self-directed interventions and complete remote assessment of objective motor and cognitive performance (eg, analysis of kinematics and reaction time) during interventions [29]. However, despite the potential, the use of serious games in VR to promote motor learning in older adults remains underexplored [30]. In addition, the generalization of skills acquired in iVR to other skills in iVR remains debated.

A recent review has proposed intriguing methods to enhance the comprehension of motor learning in VR, including tracking participants’ kinematics, manipulating sensorial feedback and difficulty parameters, and precisely simulating VR physics [27]. Prior work showed that kinematic indexes acquired in iVR (eg, movement linearity) were reliable and could possibly differentiate hand movements between healthy older adults and those with major neurocognitive disorders [31]. Additionally, several studies have provided evidence to support the idea that the provisioning of haptic and visual feedback in VR positively influenced movement smoothness, accuracy, and rapidity, thereby contributing to the improvement of motor learning in VR [32-35]. Recent evidence also highlights the efficacy of incorporating bimanual tasks in VR for promoting unimanual motor learning, aligning with the notion that the acquisition of motor skills in a digital environment can be optimized when the tasks closely mimic the complexities of everyday activities [36-38]. In line with the Yerkes-Dodson law, there is also evidence indicating that to maintain participants' motivation, the level of difficulty should be optimally balanced, neither too hard nor too easy [39]. To achieve this optimal balance, research suggests that game difficulty should be adjustable and tailored to individual participants’ motor and cognitive performance [29,40,41]. Research supports the idea that self-adaptive training, where the difficulty is adjusted based on real-time performance, can optimize learning [42]. For instance, 1 study has highlighted that individualized VR training for driving led to more effective learning and retention of performance compared to traditional VR, video, and manual training [43]. The literature indicates success rates ranging from 60% to 80% as ideal for effectively enhancing participants’ motivation [40,44].

Regarding older adults, several studies have demonstrated that motor learning in VR can be effective, resulting in improvements in motor performance along with the retention of learned skills over time. For example, a longitudinal study found that both healthy older adults and those with Parkinson disease were able to achieve learning and retention of skills across 10 different niVR games, with effective transfer of these skills to similar untrained tasks [45]. While these results are promising for niVR, the retention of iVR skills and transfer to other tasks remains underexplored. A recent multicentric large parallel randomized controlled trial (n=293) has nevertheless produced encouraging results, demonstrating that progressive cognitive-motor training in iVR was effective in older adults, with greater improvements in global cognition and physical frailty compared to traditional interventions [46].

Following current recommendations aiming at improving motor learning in older adults, we developed a self-adaptive serious game in iVR (REAsmash-iVR) [47]. This game consists of finding and hitting a digital mole as fast as possible when presented with different types of distractors. In this version, we use a regulator to continuously adapt exercise difficulty according to participants’ performance. As this version has not yet been tested among older adults, this work aims to test the feasibility and effectiveness of REAsmash-iVR in promoting motor learning within this population. Specifically, we sought to determine the transfer and retention of motor learning achieved through REAsmash-iVR to other iVR tasks. We hypothesized that REAsmash-iVR would significantly improve participants’ unimanual reaching velocity and accuracy in other iVR tasks. We also aimed to assess the effect of REAsmash-iVR on participants’ simple reaction time.

## Methods

### Study Design

This study used a short-term longitudinal pre-post design, with data collected at 3 time points: baseline (T0), immediate postintervention (T1), and 1-week follow-up (T2). This design allowed us to assess both immediate and retained motor learning effects, aligning with established motor learning literature for intermediate-term retention and transfer [39].

### Ethical Considerations

The study was conducted in adherence to the principles of the Helsinki Declaration and received approval from the Hospital-Faculty Ethics Committee of Saint-Luc-UCLouvain in Belgium (B403201524184) and the Recherche Sectorielle en Réadaptation et Intégration Sociale Ethics Committee in Canada (#2020-1909). Prior to commencing the trial, all participants provided written informed consent. The study adhered to Transparent Reporting of Evaluations with Nonrandomized Designs (TREND) guidelines. Participation was voluntary and uncompensated, and all data was collected, stored, and analyzed in a manner that ensured participant anonymity.

### Participants

The study recruited participants from the Belgian and Canadian populations between October 2022 and December 2023 using convenient sampling, leveraging word of mouth, and community outreach strategies. Inclusion criteria were individuals aged 65 years and older, possessing corrected-to-normal vision, and demonstrating the ability to comprehend simple instructions. Older adults with orthopedic or neurological disorders that might have impacted their capacity to handle a controller or that could alter upper extremity movements were excluded from the study. Participants’ cognition was screened using the Montreal Cognitive Assessment [48].

A flowchart diagram illustrating the participant flow through each stage of the study is presented in the Results section, as recommended by reporting guidelines such as TREND and CONSORT (Consolidated Standards of Reporting Trials) to ensure transparency and clarity in reporting [49,50].

### Materials

**Figure 1:**
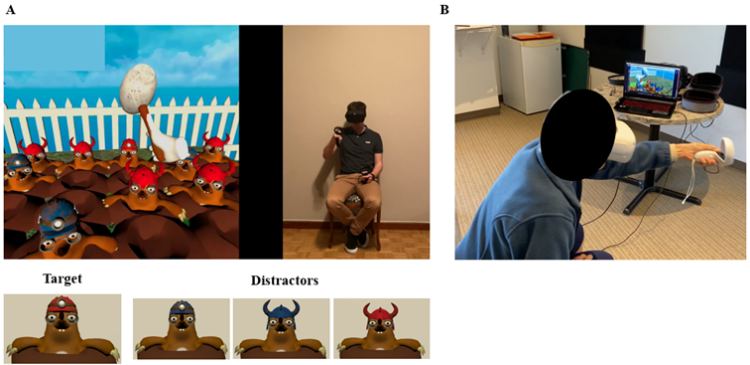
Illustration of the REAsmash-iVR. (A) The upper part of the panel simultaneously depicts the REAsmash-iVR environment as seen through the VR headset (left) and the corresponding movements performed by a participant while interacting with the system (right). The lower part of the panel shows the target (a mole wearing a red miner helmet) and the distractors (moles wearing blue miner helmets, blue-horned helmets, and red-horned helmets). (B) Experimental setup: This panel illustrates a participant playing REAsmash-iVR using an immersive VR headset connected to a computer, which streams the application. iVR: immersive virtual reality; VR: virtual reality.

The self-adaptive serious game REAsmash-iVR was developed using Unity 2019.3.15 software on the Oculus Quest 2 (Meta). This headset provides a high-resolution display (1832×1920 pixels per eye) and up to 90 Hz refresh rate, which ensures smooth and immersive interaction. The system includes 6 degrees of freedom tracking through integrated sensors, which allows participants to move freely within the digital environment. The device is equipped with 2 handheld controllers with motion tracking, which participants use to interact with the game, particularly for striking the target with a digital hammer. The controllers are equipped with motion sensors and buttons for precise input. As presented in Figure 1, Sidequest software was used to facilitate synchronization and video sharing from the headset to a laptop during the experiment.

In the REAsmash-iVR serious game, the participant is asked to locate a target as quickly as possible. The target consists of a mole wearing a red miner’s helmet and is presented among distractors (moles wearing helmets of different shapes and colors: a blue miner’s helmet, a horned blue helmet, and a horned red helmet). These distractors manipulate cognitive difficulty by competing for attention during the task, requiring participants to focus and filter out irrelevant stimuli while searching for the target. To enhance cognitive engagement, distractors were designed to vary in salience, with some mimicking the target more closely in shape and color. Throughout the game, participants were instructed to only hit the target mole with the red miner’s helmet, a task that requires both attention and precise motor action. To this end, participants used digital hammers, operated by the game controllers [47]. Motor function difficulty was manipulated through iterative practice of upper extremity reaching motions in different directions and with different levels of velocity.

In the version used here, the REAsmash-iVR used a regulator to adjust difficulty automatically and progressively based on the participants’ motor and cognitive performance. The regulator of REAsmash-iVR difficulty aims to have the participant an average 75% successful performance. As the user improved and learned, the game progressively became more difficult, thereby maintaining the 75% optimal success rate. From a motor learning perspective, as the game escalated in difficulty, participants were compelled to execute a greater number of reaching movements toward the moles, spanning further distances, and within shorter timeframes, necessitating more efficient and precise upper extremity actions.

**Figure 2:**
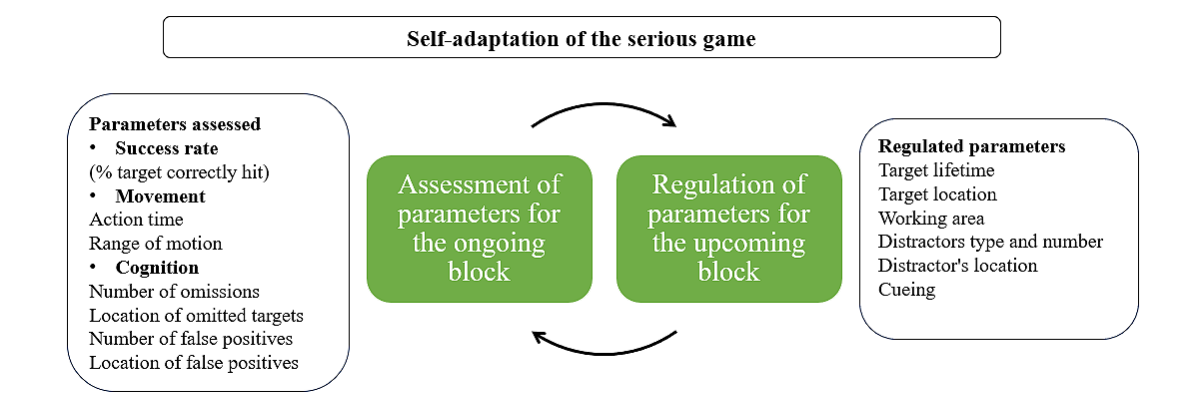
Self-adaptation of REAsmash-iVR. The system assesses performance parameters (success rate, movement, and cognition) after each block and regulates the task difficulty for the subsequent block by adjusting target and distractor characteristics, working area, and cueing to maintain an optimal challenge level for the participant. iVR: immersive virtual reality.

To ensure a continuous and progressive adaptation of the game difficulty, we used a dynamic regulation, with an infinite number of trial blocks. Each block involved finding and hitting a total of 1 to 24 target mole (trials), depending on the participant’s level of performance. Between each block, the algorithm moderates the difficulty of the game based on the overall success rate (the ratio between the number of target moles accurately hit and the number of trials) of the prior block. If the success rate was superior to 75%, the game was considered too easy by the algorithm. If the success rate ranged between 50% and 75%, the game was considered difficult. If the success rate was below 50%, the game was considered excessively difficult. Depending on the success rate of the prior block (>75% vs 50%-75% vs <50%), the parameters that were considered responsible for the observed success rate were adjusted by the algorithm. More specifically, as presented in Figure 2, during each block, in addition to the overall success rate, the algorithm evaluated the proportion of omissions (instances when the target mole is not hit vs hit), the location of omitted moles, the number of false positives (distractor moles that were hit), and the location of distractor moles hit. These outcomes were used as indicators by the algorithm to see which of the following parameters had to be adjusted for the next block: the timing and location of the target appearance, the quantity (number) and types of distractors (high vs low salience contrast), the working area (where target and distractors appeared) and the delivery of cues that helped the participant to find the target (no cues, spatial auditive cue, and visual cues).

### Experimental Protocol

Participants were tasked with engaging in REAsmash-iVR for 7 consecutive days for at least 15 minutes per day at their home. They were required to maintain a seated position throughout the gameplay and were asked to use both hands during the game. The feasibility of using REAsmash-iVR in older adults encompassed the documentation of adverse events, intervention duration (measured by the total minutes of active interaction with REAsmash-iVR, excluding pauses and time spent on menus and settings), and the evaluation of the game regulation efficacy.

To investigate whether participants’ motor learning in REAsmash-iVR transferred to other iVR tasks and demonstrate retention of this transfer, the following measures were performed before the intervention (T0), immediately after (T1), and 7 days later (T2). We assessed motor learning transfer using 2 distinct tools: the KinematicsVR and the iVR version of the Box and Block Test (BBT-VR). These tests were administered by assessors with a background in physiotherapy and experience in using VR. Motor learning transfer refers to the ability to apply skills and improvements acquired during training to new, untrained tasks or contexts. Effective transfer indicates that participants have not only improved performance in the trained task (eg, REAsmash-iVR) but also developed adaptable motor strategies that can be used in other scenarios (eg, KinematicsVR and BBT-VR).

The 1-week duration was chosen as it is commonly used in motor learning literature to assess intermediate-term retention and transfer [39,51]. This timeframe is long enough to observe whether improvements persist beyond the immediate training environment but avoids confounding factors associated with longer intervals, such as unrelated learning or natural recovery. Prior studies have similarly used 1-week retention tests to evaluate skill stabilization and generalization in both laboratory and applied contexts [39,51].

We used the KinematicsVR to assess motor learning transfer in terms of unilateral reaching performance in a novel visuo-motor task [31]. The KinematicsVR requires using one of the VR headset controllers to swiftly and precisely draw 3D shapes (straight lines and a circle) visually presented in an iVR environment. The 3D positions of the controller were registered as an export file (.csv) in the hardware of the headset and analyzed offline. This test was deemed reliable and usable to assess upper extremity kinematics (especially during the drawing of straight lines and circles) in older adults with and without major neurocognitive disorder [31]. For this protocol, participants were asked to perform the movements with their dominant hand. All participants underwent familiarization trials before the assessment. To ensure that the differences between T0 and T1 primarily resulted from the REAsmash-iVR intervention rather than a general learning effect across trials, we contrasted the changes in our sample with those observed in a previous study [31] where an equal sample of older adults underwent the test twice consecutively (with no intervention in between). Transfer in this context indicates that the REAsmash-iVR intervention contributed to general improvements in unilateral reaching performance in a novel task (KinematicsVR). The performance in Kinematics-VR was analyzed based on metrics such as movement velocity, accuracy, and smoothness, allowing us to determine the extent to which the trained skills carried over to this analytic task.

We also used the BBT-VR to evaluate motor learning transfer in terms of gross unilateral manual dexterity [52,53]. This test involved moving digital cubes one at a time from 1 side of a box to the other within a 60-second timeframe. During the test, participants were required to grasp the cubes using their thumb, index, and middle fingers while pressing corresponding buttons on the controller. The BBT-VR was found to be valid, reliable, and usable to assess manual dexterity in healthy adults and individuals with stroke [52,53]. Transfer in this context indicates that the REAsmash-iVR intervention contributed to general improvements in fine motor control and hand-eye coordination. The performance in BBT-VR was analyzed based on metrics such as the number of blocks transferred, allowing us to determine the extent to which the trained skills carried over to this dexterity-focused task.

We also evaluated the participants’ motor learning transfer to simple reaction time. The task involved detecting, as quickly as possible, a stimulus presented on a computer screen. Participants were instructed to click as quickly as possible on the touchpad when the stimuli were presented [54].

### Kinematic Analyses

By analyzing the kinematic features of movements in KinematicsVR, we could assess whether the movement strategies developed during REAsmash-iVR translated into improved performance in a new visuomotor task.

The 3D positions of the controller obtained during the Kinematics-VR test were extracted from export files (.csv) at a sampling rate of 60 Hz. The analysis of kinematic data was then conducted using a program internally developed in Python (Python Software Foundation). For each participant, a preliminary visual analysis of the data was performed (to ensure that the data were correctly acquired) and signal smoothing was applied using a Butterworth filter (sampling frequency=60 Hz; cutoff frequency=10 Hz). The following kinematic indexes were calculated: the speed-accuracy trade-off (SAT) and the spectral arc length (SPARC).

The SAT, measured in arbitrary units, is a fundamental motor learning metric that quantifies the balance between movement speed and precision, often reflecting the extent to which participants prioritize speed over accuracy or vice versa during task execution. It is widely used to assess training-induced improvements in motor performance, as optimized performance behavior tends to achieve a more favorable balance between these competing demands [55]. In this study, we computed SAT as a ratio between speed and error, using the following equation.



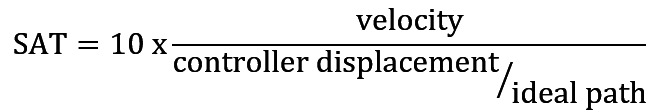



Velocity refers to the first derivative of controller position. Error is measured based on movement linearity, which involves comparing the displacement of the controller with the ideal path. A higher SAT thus reflects a more efficient balance of speed and accuracy, indicating potential motor learning gains.

SPARC is a measure of movement smoothness, which is a key aspect of movement quality and skillful performance. Smoothness, as computed with SPARC, provides critical insights into whether a movement is natural and healthy or involves compensatory strategies. Natural and healthy movements are generally smoother, reflecting efficient neuromotor control, while compensatory movements tend to be less smooth and more erratic. SPARC is computed as the arc length of the instantaneous speed spectrum (ie, the length of the curve depicting the normalized amplitude of the “speed” signal 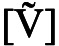 as a function of its frequency [ω]) [56]. A smoother movement involves less intermittency (alternance of acceleration and deceleration) typically resulting in a more compact and less erratic speed spectrum, leading to a small arc length. A negative sign is added to the computed arc length such a more negative SPARC value corresponds to a less smooth movement. This convention ensures that higher (less negative) SPARC values indicate smoother and more skillful motor performance.

### Data Analysis

We performed statistical analyses using Sigmaplot (version; 13.0, Systat Software Inc) with α=.05. For each analysis, we explicitly tested the normality of the data using the Shapiro-Wilk test, and the results informed the selection of appropriate statistical methods (parametric or nonparametric). As this study is the first to test the feasibility and effectiveness of REAsmash-iVR in promoting motor learning, a convenience sample of 20 participants was determined.

To evaluate the efficacy of REAsmash-iVR self-regulation, we reported the percentage of instances where participants achieved a median success rate falling within the range of 60%-80% (a range deemed acceptable for enhancing motivation in a gamified learning context [40,44]). This evaluation was performed across the initial 55 blocks (which represented the minimum number of blocks observed in all participants).

Our primary outcome was to assess participants’ motor learning transfer (and retention) to unilateral reaching performance in iVR. To analyze this, we used separate 1-way repeated measure ANOVA (or Friedman test for nonnormal data) for each shape used in the KinematicsVR assessment, comparing participants’ SAT across 3 time points: before the intervention (T0), immediately after (T1), and at follow-up (T2). Post hoc pairwise comparisons were performed to detect changes between T0, T1, and T2. We used Bonferroni or Tukey adjustments (depending on the normality of the data) to control for the increased risk of type I error due to multiple comparisons. To ensure that the observed changes in KinematicsVR metrics between T0 and T1 were predominantly attributable to the REAsmash-iVR intervention and not merely a general learning effect over trials, we compared these changes with data from a previous study [31] where older adult participants underwent the test twice consecutively without any intervention in between [31]. To compare the T1-T0 changes between this study and our prior one, we used either the Mann-Whitney rank sum test (for nonnormal data) or the 2-tailed *t* test (for normally distributed data), depending on the distribution of the data.

Secondary outcomes included the assessment of REAsmash-iVR motor learning transfer to movement smoothness, manual dexterity performance in iVR, and simple reaction time. These outcomes were analyzed using 1-way repeated measures ANOVAs (or Friedman tests), with post-hoc pairwise comparisons. We also used Bonferroni or Tukey adjustments, depending on the normality of the data.

The effect size was computed using η² for ANOVAs, ε² for the Friedman test, and adjusted Cohen *d* for parametric post-hoc pairwise comparisons and 2-tailed *t* tests. For nonparametric tests, Cohen *r* was used. η² for ANOVAs and ε² for the Friedman test provide an estimate of the effect's magnitude relative to the total variance, with values of 0.01, 0.06, and 0.14 indicating small, medium, and large effects, respectively. Cohen *d* was used to quantify the standardized mean difference between conditions, with values of 0.2, 0.5, and 0.8 representing small, medium, and large effect sizes. For pairwise comparisons following the Friedman test and between-group comparisons with the Mann-Whitney *U* test, Cohen *r* was interpreted as small (*r*≈0.1), medium (*r*≈0.3), and large (*r*≈0.5).

## Results

### Overview

**Figure 3:**
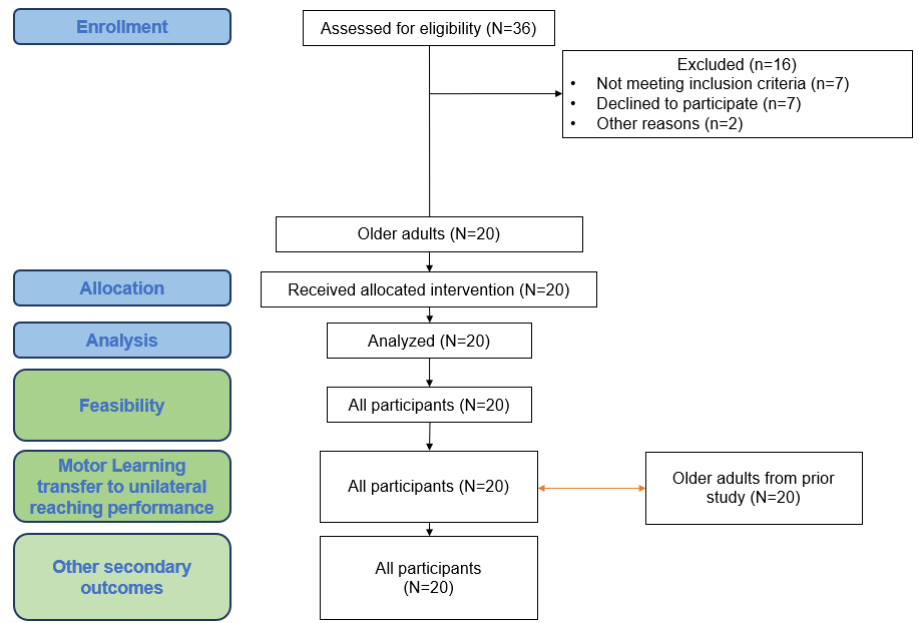
The flowchart diagram outlines the study process, including the assessment of 36 older adults for eligibility. Of these, 16 were excluded (7 did not meet the criteria, 7 declined, and 2 for other reasons). Twenty participants received the intervention, completed it, and were analyzed for feasibility, motor learning transfer, and other outcomes. Data were also compared with 20 older adult participants of a previous study.

A total of 20 older adults (of which 9 were women) with a mean age of 77.4 (SD 6.51) years participated in the study. Most of them were right-handed (n=17; 85%). A flowchart diagram is presented in Figure 3. Complementary information on participants’ characteristics is provided in Table 1.

**Table 1:** Participants’ demographics.

Characteristic		Value
Age (years), median (IQR)		77.4 (69 to 89)
**Sex, n (%)**		
	Female	9 (45)
	Male	11 (55)
**Dominant hand, n (%)**		
	Right	17 (85)
	Left	3 (15)
Height (cm), mean (SD)		169.4 (7.73)
Weight (kg), mean (SD)		77.3 (14.40)
MoCA^a^, median (IQR)		25 (6 to 30)
**KinematicsVR: Straight lines**		
	Baseline SAT^b^, mean (SD)	2.6 (1.09)
	Baseline SPARC^c^, median (IQR)	–1.97 (–2.027 to –1.896)
**KinematicsVR: Circles**		
	Baseline SAT, mean (SD)	3.2 (1.51)
	Baseline SPARC, mean (SD)	–4.45 (2.227)
	Baseline BBT-VR^d^, mean (SD)	29 (13.2)

^a^MoCA: Montreal Cognitive Assessment.

^b^SAT: speed-accuracy trade-off.

^c^SPARC: spectral arc length.

^d^BBT-VR: immersive virtual reality version of the Box and Block Test.

### Feasibility

All participants finalized the study, and no adverse event occurred during the intervention. Participants actively played with REAsmash-iVR for 7 consecutive days for a median duration of 15.8 (IQR 9.73-15.12) minutes per day.

**Figure 4:**
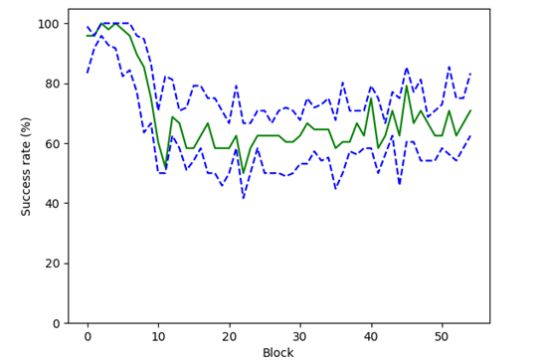
Evolution of success rate over the blocks. The x-axis represents the block number of the REAsmash-iVR intervention. The y-axis represents the participants’ motor success rate. The green line represents the median and the blue lines the 1st and 3rd quartiles issued from all participants. iVR: immersive virtual reality; VR: virtual reality.

As illustrated in Figure 4, participants’ median success rate reached a satisfactory value (60%-80%) after completing 11 blocks. Between the 11th and the 55th blocks, participants maintained a median success rate within the 60 to 80% range for 74% of the time (33 out of 44 blocks). Notably, during the final 11 blocks, participants consistently upheld a median success rate between 60% to 80%, reaching 100% coverage.

### Primary Outcome: REAsmash-iVR Motor Learning Transfer to iVR Unilateral Speed-Accuracy Performance

**Figure 5:**
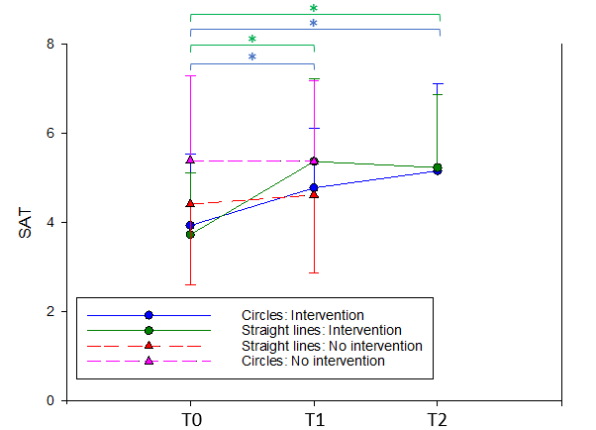
Evolution of SAT over time. The * indicates the statistical significance of the ANOVA pairwise comparisons; Results are presented as mean and SDs. SAT: speed-accuracy trade-off.

As presented in Table 2 and Figure 5, separate repeated measures ANOVA (*F*_2,38_=21.9; *P*<.001; η^2^=0.535) and pairwise comparison revealed that directly after the REAsmash-iVR intervention, participants significantly improved their SAT in drawing straight lines (t_19_=5.97; *P*<.001; Cohen *d*=1.20) with retention at T2 (T0 vs T2: t_19_=5.46; *P*<.001; Cohen *d*=1.13). Regarding the drawing of circles, participants showed significant SAT improvements (*F*_2,38_=7.7; *P*=.002; η^2^=0.290) between T0 and T1 (T0 vs T1: t_19_=2.64; *P*=.036; Cohen *d*=0.613) with retention at T2 (T0 vs T2: t_19_=3.84; *P*=.001; Cohen *d*=0.787; Table 2 and Figure 5).

**Table 2:** Speed-accuracy trade-off changes over time.

Separate ANOVAs	T0	T1	T2	*F* test (*df*)	RM^a^ ANOVA *P* value	Post hoc T0 versus T1 *P* value	Post hoc T0 versus T2 *P* value
Straight lines, mean (SD)	3.7 (1.38)	5.4 (1.85)	5.2 (1.63)	21.9 (2,38)	<.001	<.001	<.001
Circles, mean (SD)	3.9 (1.60)	4.8 (1.34)	5.2 (1.95)	7.7 (2,38)	.002	.036	.001

^a^RM: repeated measures.

When drawing straight lines, participants’ SAT improvements between T0 and T1 were found to be significantly greater than those observed in participants who did not follow any intervention between the tests (t_19_=3.0; *P*=.005; Cohen *d*=0.487). Contrastingly, when drawing circles, participants’ SAT improvements between T0 and T1 were not significantly greater than those observed in participants who did not follow any intervention between the tests (t_19_=1.96; *P*=.060; Cohen *d*=0.318).

### Secondary Outcomes: REAsmash Motor Learning Transfer to iVR Movement Smoothness

As presented in Table 3, the Friedman test and pairwise comparisons showed that for the drawing of straight lines, participants did not observe significant SPARC changes between T0, T1, and T2 (*χ*²_2_=2.1; *P*=.35; ε^2^=0.003; Table 3).

**Table 3:** Movement smoothness changes over time.

Separate ANOVAs	T0	T1	T2	Chi-square (*df*)	Friedman test *P* value	Post hoc T0 versus T1 *P* value	Post hoc T0 versus T2 *P* value
Straight lines, median (IQR)	–1.97 (–2.027 to –1.896)	–1.92 (–1.978 to –1.783)	–1.91 (–2.016 to –1.811)	2.1 (2)	.35	N/A^a^	N/A
Circles, median (IQR)	–3.77 (–5.876 to –2.517)	–2.67 (–4.000 to –2.359)	–2.44 (–3.287 to –2.273)	11.2 (2)	.004	.03	.004

^a^N/A: not applicable.

Contrastingly, for the drawing of circles, participants showed significant SPARC improvements (*χ*²_2_=11.20; *P*=.004; ε^2^=0.23) between T0 and T1 (*q*=3.58; *P*=.03; Cohen *r*=0.8), with retention at T2 (T0 vs T2: *q*=4.47; *P*=.004; Cohen *r*=1.0; Table 3). Moreover, improvements between T0 and T1 were significantly greater than those observed in participants who did not follow any intervention between the tests (t_19_=2.41; *P*=0.010; Cohen *d*=0.381).

### Secondary Outcomes: REAsmash Motor Learning Transfer to Manual Dexterity Performance

As presented in Table 4, the Friedman test (*χ*²_2_=25.9; df=2; *P*<.001; ε²=0.532) and pairwise comparison revealed that participants significantly improved their overall BBT-VR score between T0 and T1 (*q*=5.26; *P*<.001; Cohen *r*=0.678) with significant retention at T2 (T0 vs T2: *q*=6.82; *P*<.001; Cohen *r*=0.880).

**Table 4:** BBT-VR^a^ scores and reaction times change over time.

	T0	T1	T2	Statistics	RM^b^ ANOVA or Friedman test *P* value	Post hoc T0 versus T1 *P* value	Post hoc T0 versus T2 *P* value
BBT-VR score (blocks), median (IQR)	27 (20.1 to 36.4)	36 (29.8 to 45.1)	38 (29.7 to 50.5)	*χ*^2^_2_=25.9	<.001	<.001	<.001
Simple reaction time (ms), mean (SD)	398.1 (116.89)	399.9 (94.12)	379.6 (80.31)	*F*_2,38_=0.5	.64	N/A^c^	N/A

^a^BBT-VR: immersive virtual reality version of the Box and Block Test.

^b^RM: repeated measures.

^c^N/A: not applicable.

### Secondary Outcomes: REAsmash Effect on Simple Reaction Time

Participants did not show significant simple reaction time changes (*F*_2,38_=0.5; *P*=.64; η^2^=0.024) between T0, T1, and T2 (Table 4).

## Discussion

### Principal Findings

This short-term longitudinal pre-post study aimed to evaluate the feasibility and effectiveness of using a self-adaptive serious game, REAsmash-iVR, to enhance motor learning and simple reaction time in older adults. Our results suggest that, on average, participants required 10 to 40 blocks to benefit from an acceptable-to-optimal level of success rate when starting from the easiest level of difficulty (block 0). Moreover, outcomes indicated that a 7-day intervention with REAsmash-iVR resulted in improved performance in other iVR tasks, as evidenced by enhanced SAT metrics in drawing straight lines and circles, and increased score in displacing digital blocks postintervention. Notably, significant retention of speed/accuracy improvement was observed at the 1-week follow-up for the drawing of straight lines and circles, and for the displacement of digital blocks. Similarly, secondary analyses revealed that the REAsmash-iVR intervention led to improved movement smoothness in drawing circles but not straight lines. Finally, we did not observe any significant effect of REAsmash-iVR on simple reaction time.

### REAsmash-iVR Impact on Motor Learning

Our findings seem to indicate that older adults effectively achieved motor learning. After undergoing the REAsmash-iVR intervention, participants exhibited the ability to apply their skills in various contexts, as evidenced by enhanced speed and accuracy in KinematicsVR and improved performance in BBT-VR. Notably, certain improvements persisted even 1 week after the intervention, suggesting intermediate-term retention of learned skills and their potential transfer to other tasks. These findings may highlight a meaningful step in motor learning within this time frame.

On the one hand, our results [31] may indicate that the notable enhancements in speed-accuracy when drawing straight lines postintervention could be credited to the REAsmash-iVR intervention, rather than being merely a result of general learning or increased familiarity with the device and setup. In fact, in our prior study [31], where no intervention occurred between assessments, we observed no significant changes in SAT in KinematicsVR tasks, further supporting the conclusion that the improvements in this study are specifically attributable to the REAsmash-iVR intervention. The self-adaptive nature of REAsmash-iVR, which allowed for tailored and optimized adjustments to accommodate each participant’s unique needs and capacities, likely played a role in optimizing engagement and facilitating skill acquisition, ultimately leading to the observed enhancements in motor performance. Previous research has demonstrated that older adults achieved significant motor learning and skill acquisition when provided with appropriate optimized interventions tailored to their specific needs and abilities. For instance, a study comparing a group who practiced a square-stepping task with enhanced feedback, autonomy-supportive choices, and optimized instructions to a control group practicing without these elements, found that the experimental group exhibited faster movement times during both practice and retention phases [57]. Similarly, feedback from participants in our study overwhelmingly attested to their enjoyment of the game. Many remarked on the engaging nature of the REAsmash-iVR intervention, highlighting its immersive qualities and the satisfaction derived from mastering new skills within the digital environment. Furthermore, studies have emphasized the importance of leveraging technological advancements to develop innovative interventions that cater to the unique challenges and preferences of older adults [58,59]. In a prior study, researchers showed that an adaptive video game training intervention led to generalized positive effects on cognitive control abilities in older adults [59]. Our findings also align with [45] a longitudinal, controlled clinical study investigating motor learning, retention, and transfer in older adults using VR-based training, specifically focusing on individuals with Parkinson disease [45]. Their results demonstrated that older adults with Parkinson disease could effectively learn new motor skills through VR-based interventions. Importantly, the study observed improvements not only in motor performance but also in the retention and transfer of learned skills to real-world tasks.

The greater performance improvement here, relative to our previous study [31] could be attributed to the participants exhibiting lower baseline performance levels in this study, potentially allowing for a greater margin of improvement. This difference becomes particularly apparent when considering circle drawing tasks, where participants had similar baseline performance levels, and improvements in speed and accuracy did not exceed those seen in our prior study [31]. However, this divergence in outcomes could also stem from the inherent differences between drawing straight lines and circles (straight vs cyclic movements). Notably, it could be hypothesized that due to the similarity in movement nature (discrete reaching movements) between drawing straight lines and hitting digital moles, REAsmash-iVR likely played a significant role in augmenting speed and accuracy outcomes in straight-line drawing tasks.

### Disparities in Transfer to Movement Smoothness and Manual Dexterity in iVR

Secondary analyses revealed that participants demonstrated enhanced movement smoothness in drawing circles but not straight lines. In a prior study, researchers have recently indicated that the intermittency of movement, as evidenced by the number of velocity peaks, is influenced by the specific task being performed in older adults [60]. In our study, the lack of significant changes observed in the drawing of straight lines could potentially be attributed to the 2D and discrete nature of these movements within the KinematicsVR application. This limitation may have restricted the scope for enhancement. Conversely, when considering the drawing of circles in KinematicsVR, the continuous and 3D nature of the movements offers potential for improvement in an additional dimension (compared to the drawing of straight lines where only 2 dimensions are considered in the KinematicsVR assessment). These hypotheses are supported by the baseline results, wherein participants demonstrated a median SPARC score of –1.97 (IQR –2.027 to –1.896) for straight lines, compared to a median SPARC score of –3.77 (IQR –5.876 to –2.517) for circles. Due to the tendency for smoother movements to be indicated by SPARC values closer to 0, these results underscore a heightened potential for enhancement in circle drawing tasks.

### REAsmash-iVR Effect on Simple Reaction Time

The study did not observe any significant effect of REAsmash-iVR on simple reaction time. Although these findings could have been expected, they may reflect the specific design limitations of the intervention and assessment or the need for longer intervention durations to detect changes in these outcomes. The task in iVR involved locating and responding to digital moles as quickly as possible, suggesting the potential for improvements in simple reaction times. Especially since our sample had a mean reaction time (mean 398.1, SD 116.89 ms) more than the normal in equally aged standards [54]. In comparison, in a prior study [54], researchers observed that the average mean reaction time of individuals aged between 61 and 80 years for the same task was 296.1 (SD 63.9) milliseconds. However, REAsmash-iVR engages spatial attention and distractor inhibition, aspects that cannot be adequately assessed solely through simple reaction time measurements, as the latter primarily evaluates alertness levels. Moreover, it is important to consider that reaction time improvements tend to be modest in older adults due to age-related declines in neurological processing speed [61]. Several studies have documented a decline in reaction times with increasing age, reflecting changes in neurological networks and cognitive processing abilities [62-64]. A recent study assessing 861 participants aged 70-90 years also observed that an increase in intraindividual variability of reaction time, considered as a cognitive marker of neurobiological disturbance, was associated with dementia and mortality [65]. Therefore, longer intervention periods and more comprehensive assessments may be necessary to capture subtle improvements in reaction times among older adults participating in VR-based interventions.

### Limitations

We acknowledge the following limitations. First, the study’s sample size (n=20) and design (pre-post), while appropriate for initial exploration, pose limitations to the generalizability and robustness of the findings. Although a retrospective comparison was used to evaluate the effect of REAsmash-iVR on motor learning, using a randomized controlled trial design would provide stronger evidence for drawing conclusions regarding the intervention's efficacy.

Second, the length of the intervention period may have been too short to observe significant improvements in certain outcomes (eg, reaction time), particularly among healthy participants. A longer study duration would be beneficial for capturing more substantial changes. Future investigations involving healthy older adults and individuals with major neurocognitive conditions could provide valuable insights into the effects of REAsmash-iVR on a broader range of participants.

Third, for the BBT-VR, retrospective comparisons between participants who received the REAsmash-iVR intervention and those who did not were possible. As a result, it remains challenging to definitively attribute observed improvements solely to the intervention itself, as opposed to potential learning effects or mere familiarization with the VR device and testing procedures. Especially since the minimal detectable change of the BBT-VR in healthy adults is relatively high (14.06 for the dominant hand and 18.23 for the nondominant hand) [52]. Therefore, future studies incorporating appropriate control groups are essential to establish the causal relationship between the REAsmash-iVR intervention and the observed transfer of performance.

### Implications

The results of this study carry significant clinical and research implications. Clinically, they underscore the potential of VR technology as a novel and engaging approach to promote motor learning and rehabilitation in older adults. Previous research has demonstrated that VR-based interventions can improve motor function and engagement in rehabilitation through gamified experiences, particularly in older populations with age-related declines [66-69]. Health care practitioners working in rehabilitation settings may consider integrating VR-based interventions into their programs targeting motor impairments and age-related declines in physical function. More importantly, the use of self-adaptive serious games such as REAsmash-iVR may offer participants the opportunity for self-rehabilitation through tailored interventions. These interventions can be specifically designed to target individual motor and cognitive deficits, providing a personalized approach to rehabilitation. Personalization in VR rehabilitation has been shown to improve outcomes by adapting difficulty levels based on real-time performance data [43]. Furthermore, the diverse range of applications available within the VR headset may enable participants to receive real-time feedback on their performance, allowing for continuous monitoring of progress and identifying areas for improvement. Several studies have highlighted the potential of iVR to assess relevant quantitative outcomes, such as hand kinematics, gaze tracking, and reaction time, in a valid and reliable manner [47,70-72]. While traditional assessments may suffer from ceiling or floor effects, limiting their sensitivity to subtle changes in performance, these quantitative metrics provide precise, objective insights into how participants behave during the task [72,73]. This allows clinicians and researchers to track nuanced motor and cognitive responses, facilitating more tailored rehabilitation strategies. Such feedback mechanisms could enhance motivation and engagement, facilitating more effective rehabilitation outcomes. VR may boost motivation by providing immersive environments that promote goal-oriented tasks, immediate feedback, and a sense of achievement through gamified elements [74]. These features not only increase adherence to rehabilitation programs but also foster a positive emotional response, which is critical for sustaining long-term engagement and improving functional recovery [75,76]. Additionally, with advancements in VR and mixed-reality headset technologies, iVR devices hold promise for promoting social interaction and connectivity among older adults [77,78]. Research has shown that digital environments can foster social engagement, reducing isolation and improving mental well-being [79,80]. Digital conferences, collaborative gaming experiences, and social environments can be facilitated through VR platforms, fostering social engagement and reducing feelings of isolation, which are particularly relevant in the context of aging populations and social distancing measures. Incorporating these social aspects into VR-based interventions could not only enhance the overall user experience but would also contribute to the holistic well-being of older adults.

From a research perspective, the study highlights the importance of exploring optimal parameters and mechanisms underlying VR interventions to maximize their therapeutic benefits, eventually using regulators of difficulty. Larger controlled studies are needed to elucidate the long-term effects, optimal dosage, and generalizability of VR interventions across different populations and settings.

### Conclusions

This study provides valuable insights into the feasibility and potential effectiveness of using a self-adaptive serious game to enhance motor learning in older adults. While the intervention demonstrated promising results in improving reaching accuracy and velocity balance, and movement smoothness, future research is warranted to elucidate its broader impact on physical and cognitive function in aging populations.

## Data Availability

The data that support the findings of this study are available on request from the corresponding author.

## Abbreviations

BBT-VR: immersive virtual reality version of the Box and Block Test
CONSORT: Consolidated Standards of Reporting Trials
iVR: immersive virtual reality
niVR: nonimmersive virtual reality
SAT: speed-accuracy trade-off
SPARC: spectral arc length
TREND: Transparent Reporting of Evaluations with Nonrandomized Designs
VR: virtual reality
